# An Intimate Partner Violence informational program in a hospital fracture clinic: a pre-test post-test intervention study

**DOI:** 10.5249/jivr.v9i1.793

**Published:** 2017-01

**Authors:** Investigators PREVAIL

**Affiliations:** ^a^Department of Clinical Epidemiology & Biostatistics, McMaster University, Ontario, Canada.

**Keywords:** Intimate partner violence, Intervention program, Orthopaedic trauma, Spouse abuse

## Abstract

**Background:**

Many organizations have conducted Intimate Partner Violence (IPV) informational campaigns, but the extent to which such cost-effective, simple changes to the clinic environment can improve patient perceptions about IPV is largely unknown. Our primary objective was to determine how an IPV informational program affects patients’ perceptions about discussing IPV in a fracture clinic setting.

**Methods:**

We conducted a pre-post intervention study to evaluate the impacts of an IPV informational program on patients’ perceptions and willingness to discuss IPV in an orthopaedic fracture clinic setting. During the intervention phase, there were posters and brochures in each bed area and several places in the waiting area, and the surgeons received a button to wear on their lab coat stating their openness to discuss IPV and a set of instructions on how to ask patients about IPV and refer them to resources.

**Results:**

A total of 160 patients (80 pre-intervention and 80 post-intervention) have participated in this study. Overall perception of the clinic as an open place in which to discuss IPV did not change as a result of the informational program compared to the control setting. However, more patients exposed to posters and information about IPV believed the clinic staff possessed resources to help IPV victims compared to the control group; however, this difference did not reach statistical significance (62% vs. 53%, respectively, p=0.29).

**Conclusions:**

Passive interventions may serve an adjunctive role in facilitating active interventions in a clinic environment, but should not be considered in isolation as an effective approach.

## Introduction

Intimate Partner Violence (IPV), also known as spouse abuse or domestic violence, has emerged as one of the predominant forms of violence affecting North American women today.^1^ In Canada, of the known IPV cases, 40% of women have suffered a physical injury, whereby 15% of these cases were serious enough to warrant medical attention. ^2^ A recent global prevalence study found that 1 in 6 women attending orthopaedic fracture clinics had experienced IPV in the past year and 1 in 50 women presented to clinic for treatment of an IPV-related injury. ^3^ The authors of that study argue that orthopaedic surgeons are well-positioned to identify IPV victims who have experienced serious abuse and to provide referrals to appropriate support services.

Building upon this research, we aimed to develop, implement, and evaluate a simple informational program within the MSK injury setting. Organizations have implemented similar informational campaigns with posters and/or brochures as their central focus. Such interventions have been used, for example, for smoking cessation,^4^ hand hygiene,^5^ catheter compliance^6^ and many other public health issues with varying degrees of success.

A controlled study has not previously been performed to demonstrate the efficacy of a poster and brochure-based intervention alone, this study evaluated the potential for an inexpensive way to improve perceptions of the fracture clinic as an appropriate setting to discuss IPV. Our primary objective was to determine how an IPV informational program affects patients’ perceptions about discussing IPV in a fracture clinic setting. We also aimed to explore differences between men’s and women’s perceptions about discussing IPV in a fracture clinic setting.

## Methods

**Overview**

**Figure 1:**
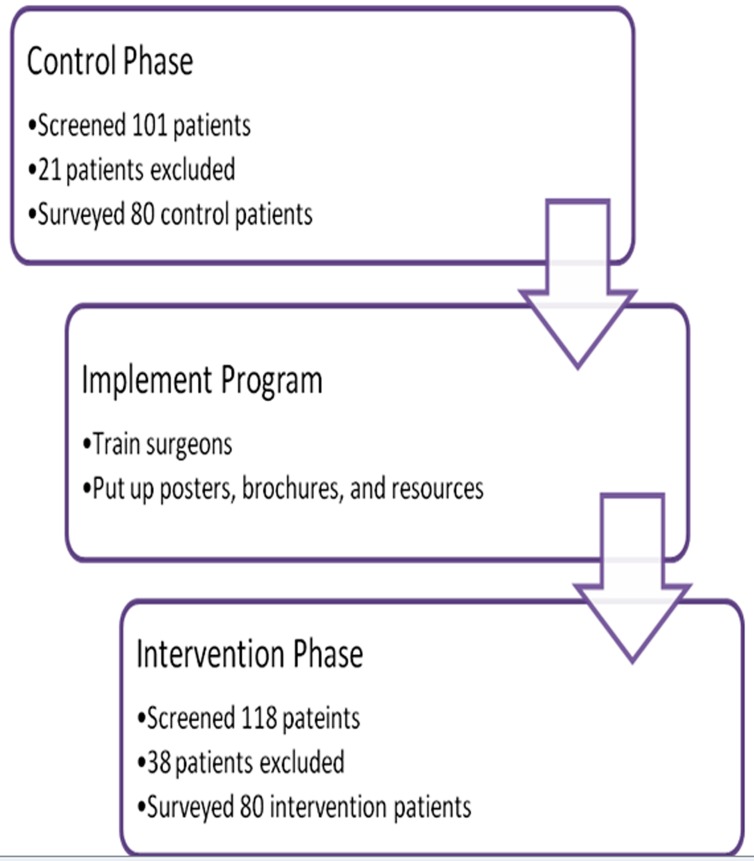
Study Flow Diagram

We conducted a pre-test/post-test intervention study at a Level I trauma center to compare patient perceptions about IPV within the setting of an orthopedic fracture clinic before and after implementation of an informational campaign. The study had 3 phases: 1) Control phase; 2) Program implementation phase; and 3) Intervention phase (Figure 1).

**Eligibility and Screening**

A study coordinator assessed eligibility of all men and women who presented to the fracture clinic. To be considered for inclusion in the study, the patient had to: 1) present to the fracture clinic for his/her own appointment; 2) be sixteen years of age or older; 3) be able to read, understand, and write in English; and 4) be able to separate him or herself from anyone who accompanied him or her to the clinic to ensure that they could complete the survey in privacy. We excluded patients if they were too ill, injured, or cognitively impaired to participate, or declined to give informed consent. We recorded the number of patients screened and reasons for ineligibility. 

**Control Phase**

The control phase was a three-week period in which the investigators administered the study questionnaire to every consenting patient. We made no changes to the clinic environment for this phase.

**IPV Information Program**

**Figure 2:**
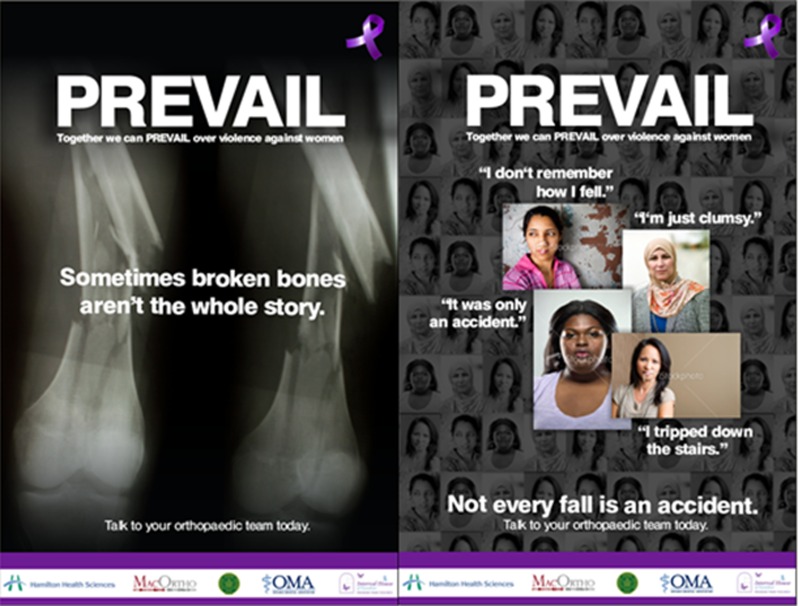
Study posters

**Figure 3:**
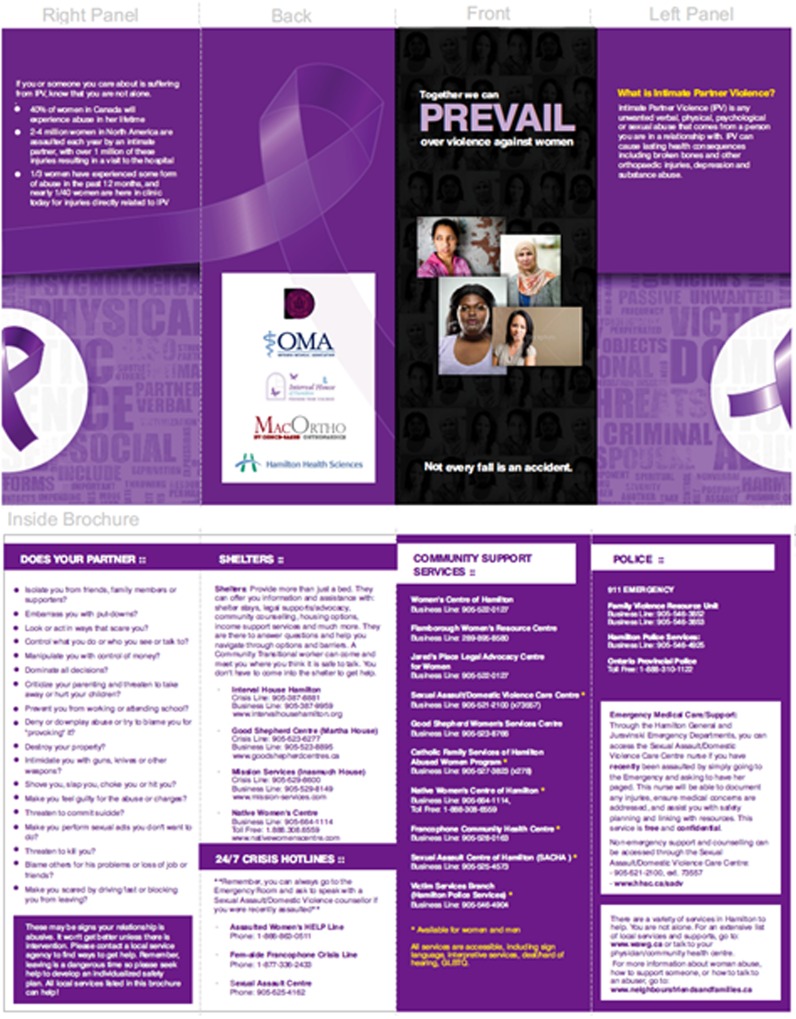
Study Brochure

Immediately following the control phase, the investigators implemented the IPV informational program. The program included displaying two versions of an orthopedics-centric IPV poster (Figure 2), an informational brochure containing local shelter, emergency and counselling resources for the Hamilton area (Figure 3), an emergency wallet card insert containing 24/7 crisis information and safety information about leaving an abusive relationship, and staff buttons demonstrating clinic awareness of IPV.

Four posters were displayed in the fracture clinic waiting area and one poster was displayed in each patient bed area for a total of nine posters. A stand containing both the informational brochure and emergency card insert was displayed on a clinic waiting room table and an additional stand was positioned in each patient bed area.

We provided a training session for fracture clinic staff on what to do if a patient discloses that they have been a victim of IPV. We provided a one page summary of the training session content which was displayed in the dictation room of the clinic. We also notified relevant local organizations so that they were prepared for a potential increase in referrals. 

**Outcome Measures and Survey**

Patients in both groups completed a self-report written questionnaire. The primary outcome of the study was to determine participant’s perception that the orthopaedic clinic is an open, interesting, safe and supportive environment in which IPV is discussed. This was measured with one question on a scale of one to ten. The secondary outcome was measured with sixteen questions using a Likert scale focusing on patients’ perception of the concept of IPV in general, and how they perceive the fracture clinic environment. 

The questionnaire contained questions on demographics, general perceptions about IPV, perceptions about IPV specific to fracture clinic settings, and one question on overall perception of the fracture clinic as an appropriate setting to discuss IPV. We did not include direct questions about posters, buttons or brochures in the questionnaire to minimize social desirability bias. The survey included questions on patients’ perceptions that were previously developed and used for assessing fracture clinic patients’ perceptions of IPV.^7^ The questions appear to meet face and content validity criteria. Methodological and IPV experts were involved in the development of the questionnaire.

**Sample Size**

With a type I error level set at 0.05 and a power of 80%, on a two-tailed test, assuming a standard deviation of two points on our primary outcome measure ten point scale, we were able to detect a 0.9 point change with 80 patients in each group. Similarly, our sample size would be able to detect a 20% difference between groups in patient perceptions about the clinic having staff and resources for IPV victims. Given that our participating surgeons see approximately 100 to 150 patients per week in their fracture clinics, we anticipated that it was feasible to recruit 160 individuals in a period of six weeks, with 80 participants allotted for the control phase and 80 for the IPV informational program phase.

**Statistical Analysis**

We present descriptive analyses, including frequency counts and percentages, for all collected data. Continuous data are presented as means and standard deviations. Patients who completed part of the questionnaire were included in our analyses. Since there were very little missing data (less than 10% in most cases), we did not complete imputations for missing information.

We compared means across IPV informational program and control groups for the primary outcome using an independent t-test. We also used chi-squared tests to compare responses across groups for the questions on perceptions of IPV in general and perceptions of the fracture clinic as a place to talk about IPV and we compared attitudes of men versus women with chi-squared tests. Significance level was set at p<0.05 for all analyses.

We performed a multivariate linear regression analysis and univariate linear regressions to determine if selected demographic characteristics were associated with patients’ overall perceptions of discussing IPV in a fracture clinic setting. We used the following predictor variables: age (continuous), ethnicity, gender, income level, education level, and whether the patient was in the IPV intervention group. All data analyses were performed using SPSS version 22.

## Results

**Characteristics of Included Participants**

160 patients (80 pre-intervention and 80 post-intervention) participated in this study (Figure 1). Most participants were Caucasian (125/160, 78%), married (59/160, 37%) with children (101/160, 63%), and in a long-term relationship (mean length 21 years) (Table 1). The mean participant age was 46 (range 17-83), with a slight predilection for female patients (84/160, 53%). The majority of patients were presenting to the clinic with a fracture (115/158, 73%). 

**Table 1:** Patient Characteristics

Characteristic	Intervention		Controls		TOTAL	
	n	%	n	%	n	%
Age mean (SD)	45 (19)		46 (17)		46 (18)	
Gender						
Female	40	50	44	55	84	53
Male	40	50	36	45	76	48
Income						
Less than $20 000	16	21	17	24	33	22
$20 000 to $40 000	18	24	12	17	30	20
$40 000 to $60 000	11	15	10	14	21	14
$60 000 to $80 000	9	12	19	26	28	19
$80 000 to $100 000	11	15	7	10	18	12
More than $100 000	10	13	7	10	17	12
Level of Education						
Did not finish high-school	13	17	13	17	26	17
High-school diploma	18	23	18	23	36	23
Post-secondary education	48	61	46	60	94	60
Ethnicity						
Caucasian	62	79	63	81	125	80
Other	17	22	15	19	32	20
Marital Status						
Married	25	31	34	43	59	37
Common law partnership	4	5	14	18	18	11
Divorced or separated	14	18	7	9	21	13
Widowed	7	9	4	5	11	7
Single	30	38	21	26	51	32
Length of Relationship (years) mean (SD)	20 (17)		23 (16)		21 (17)	
Children						
Yes	50	63	51	64	101	63
No	30	38	29	36	59	37
Sexual Orientation						
Heterosexual	77	99	73	94	150	97
Homosexual	0	0	2	3	2	1
Bisexual	1	1	2	3	3	2
Type of Injury						
Fracture	57	73	58	73	115	73
Dislocation	3	4	2	3	5	3
Arthritis	1	1	4	5	5	3
Sprain/Strain	1	1	5	6	6	4
Soft tissue problems	1	1	2	3	3	2
Unsure	3	4	1	1	4	3
Other	12	15	8	10	20	13

**Patient Perception of the Fracture Clinic Setting **

The mean score among control group participants was 6.62 (SD 2.34) out of a possible score of 10 on the question regarding the patient perception of the orthopedic environment as an open and safe environment in which to discuss IPV. The mean score among intervention group participants was 6.62 (SD 2.33) on the same ten-point scale question. The scores were not significantly different (p=0.99) across intervention and control groups.

**Patient Attitudes Toward IPV Screening and the Fracture Clinic Setting**

Patients who attended clinics after implementation of the IPV informational program trended to higher agreement that the clinic had the appropriate staff and resources to help victims of IPV (62% vs. 53%, respec-tively, p=0.29) but this was not statistically significant (Table 2). Similarly, patients in the intervention group trended toward believing that talking about IPV for victims in the clinic would help (87% vs. 79.5%, respectively, p=0.22) (Table 2). Men and women had similar attitudes on IPV screening. Only one significant difference emerged: women were significantly more likely to believe that IPV affects many people in Canada (97.6% vs. 80.8% respectively, p=0.001) (Table 3).

**Table 2:** Patient attitudes towards IPV and screening in the fracture clinic.

	Intervention		Control		P value
	n	%	n	%	
IPV is a serious health issue (N=77,79)					0.954
Strongly Agree/Agree	68	88	70	89	
Strongly Disagree/Disagree/ Neutral	9	12	9	11	
IPV affects many people in Canada (N=77,78)					0.617
Strongly Agree/Agree	70	91	69	88	
Strongly Disagree/Disagree/ Neutral	7	9	9	12	
Orthopedic surgeons care about IPV (N=76,80)					0.330
Strongly Agree/Agree	44	58	53	66	
Strongly Disagree/Disagree/ Neutral	31	41	27	33	
Orthopedic surgeons are focused only on injuries, not overall health (N=78,80)					0.887
Strongly Agree/Agree	43	55	45	56	
Strongly Disagree/Disagree/ Neutral	35	45	35	44	
Orthopedic surgeons are qualified to answer questions/offer support to victims of IPV (N=78,79)					0.809
Strongly Agree/Agree	37	47	39	49	
Strongly Disagree/Disagree/ Neutral	41	53	40	51	
The orthopedic clinic is a safe and open place to discuss IPV (N=78,80)					0.896
Strongly Agree/Agree	32	41	32	40	
Strongly Disagree/Disagree/ Neutral	46	59	48	60	
The orthopedic clinic has the right staff and resources to help victims of IPV (N=78,79)					0.289
Strongly Agree/Agree	48	62	42	53	
Strongly Disagree/Disagree/ Neutral	30	39	37	47	
Talking about IPV in the orthopedic clinic will not help (N=76,78)					0.223
Strongly Agree/Agree	10	13	16	21	
Strongly Disagree/Disagree/ Neutral	66	87	62	80	

**Table 3:** Attitudes of Men versus Women on IPV Screening in the Fracture Clinic.

	Women		Men		P value
	n	%	n	%	
IPV is a serious health issue (N=82,74)					0.617
Strongly Agree/Agree	74	90	64	87	
Strongly Disagree/Disagree/ Neutral	8	10	10	14	
IPV affects many people in Canada (N=82,73)					0.001
Strongly Agree/Agree	80	98	59	81	
Strongly Disagree/Disagree/ Neutral	2	2	14	19	
Orthopedic surgeons care about IPV (N=81,75)					0.511
Strongly Agree/Agree	53	65	45	60	
Strongly Disagree/Disagree/ Neutral	28	35	30	40	
Orthopedic surgeons are focused only on injuries, not overall health (N=83,75)					0.523
Strongly Agree/Agree	44	53	44	59	
Strongly Disagree/Disagree/ Neutral	39	47	31	41	
Orthopedic surgeons are qualified to answer questions/offer support to victims of IPV (N=83,74)					0.873
Strongly Agree/Agree	41	49	35	47	
Strongly Disagree/Disagree/ Neutral	42	51	39	53	
The orthopedic clinic is a safe and open place to discuss IPV (N=83,75)					0.421
Strongly Agree/Agree	31	37	33	44	
Strongly Disagree/Disagree/ Neutral	52	63	42	56	
The orthopedic clinic has the right staff and resources to help victims of IPV (N=83,74)					0.873
Strongly Agree/Agree	47	57	43	58	
Strongly Disagree/Disagree/ Neutral	36	43	31	42	
Talking about IPV in the orthopedic clinic will not help (N=82,72)					0.369
Strongly Agree/Agree	25	31	17	21	
Strongly Disagree/Disagree/ Neutral	57	70	55	76	

Multivariate and univariate linear regressions did not find any factors significantly associated with the overall perception of the fracture clinic as a safe environment to discuss IPV (Table 4).

**Table 4:** Linear Regression of Selected Demographic Characteristics

Characteristic	Multivariate		Univariate	
	Coefficient (95%CI)	p-value	Coefficient (95%CI)	p-value
Higher Age	0.015 (-0.006-0.036)	0.168	0.016 (-0.005-0.036)	0.131
Male gender	0.160 (-0.595-0.915)	0.676	0.187 (-0.549-0.924	0.616
Annual income below $20,000 (CAD)	-0.425 (-1.325-0.503)	0.367	-0.637 (-1.535-0.260)	0.163
No post-secondary education	-0.540 (-1.330-0.250)	0.179	-0.738 (-1.482-0.006)	0.052
Caucasian ethnicity	0.296 (-0.638-1.231)	0.532	0.369 (-0.533-1.270)	0.420
IPV toolkit intervention group	0.073 (-0.655-0.801)	0.844	Unable to assess	--

## Discussion

This is the first study, of which we are aware, that has explored the impact of an IPV informational program in orthopedic fracture clinics. We did not find that a low cost IPV informational program significantly affected patients’ overall perceptions of the fracture clinic environment. Methods implemented in other outreach campaigns and other environments have been previously researched. A study demonstrating administrative over-sight of IPV screening methods significantly enhanced the compliance of the staff indicates that perhaps a larger, more “active” program within a hospital system may provide stronger organizational support of the initiative.^8^ Our study, which relied upon the individual cooperation of the members of a small clinic, thus may not have been optimally effective, as the weight of the institution was not yet behind it. Garnering the support of whole hospital systems and bridging across specialties would represent a similar movement such as the recent move to implement osteoporosis screening programs in fracture clinics, a true success story in the integration of a classically medical problem within the surgical realm.

Strengths of this study include that we had a high survey completion rate during recruitment (87% of eli-gible patients). That our hospital has participated in numerous IPV related studies previously may be con-sidered both strength and limitation of our work. Positively, the clinic staff has been well-versed in the issue at hand for a number of years and was extremely cooperative and enthusiastic about the implementation of our informational program. This extensive experience with IPV may have affected our results in the patients’ survey responses were potentially influenced by the clinic’s history. Other limitations include the fact that some patients, upon hearing the term “domestic violence” or “intimate partner violence”, immediately assumed that they were being asked about their own personal experience with violence. Despite careful counseling and review of written study information sheets, the majority of those patients who declined to participate after the study was explained to them did so with stated reasons such as “I am not an abuser” or “my injury was not because of abuse”, indicating a potential disconnection between the study goals and participant interpretation thereof.

A possible source of bias also exists based on injury type, as many patients presenting with injuries to the hand (especially dominant hand) declined to participate because they could not currently write. More investigation would need to be done as to the specific subcategories of fractures/sprains that domestic violence victims sustain to ascertain whether or not this limitation of a written questionnaire has bearing on our results. Factors such as clinic waiting time and other scheduling frustrations also have potential influence on a survey that asks for patients' opinions. Additionally, as some patients were recruited from the waiting room and others in the patient bed areas while waiting to be seen by a resident or surgeon, different lengths of exposure to the materials may possibly influence their opinion. Lastly, issues such as whether or not the patient came to the clinic alone, and the acuity of the injury/pain level of the patient may have influenced whether and how much a patient took notice of the clinic décor and thus the potential influence those implements may impart.

Although the patients were not randomized to the IPV informational program or control groups, we believe that this study still has a robust design. Since the patients were from the same clinic, this reduced the variability within and between groups which increased our power to detect a difference between groups. We chose not to randomize the patients to the IPV informational program or control group due to the logistics of implementing the informational program. It was not feasible to conduct a multicenter cluster randomized trial because the program was developed to be implemented locally, with local resources and organizations in mind. A future direction for studies could be to develop a program that applies to a broader region and conduct a multicenter study.

In addition to developing interventions aimed at patients, more attention should be paid to physicians and allied health professional training and education to allow more active measures to create an optimal clinic environment for victims of IPV. Part of the educational process should be to reinforce that the well-being of the entire patient is the goal of every clinical encounter, not merely the treatment of musculoskeletal injuries. Positively, this study did encourage the open discourse of this important public health concern among major stakeholders in Canadian healthcare, which will hopefully make for more enthusiastic and seamless dissemination of the ideas presented here across other orthopedic centers. 

Since this informational program was designed to be taken up passively by the patients in the clinic (i.e. we made no effort to actively ensure that the patients received the materials), future research could focus on developing more active interventions for raising awareness of IPV in fracture clinics. We recommend that future studies focus on multifaceted interventions incorporating a more active distribution of materials to patients, reinforcing surgeons’ knowledge with participatory education sessions and multiple follow up training sessions, a mobile application with resources for surgeons, and a focus on evaluating the uptake and retention of study materials. Future research could also evaluate more “downstream” effects of the program, for example, a change in IPV disclosure and referral rates.

## Conclusion

While posters and brochures seem logical and helpful, they alone are insufficient to improve overall patient impressions of the fracture clinic environment. They may serve an adjunctive role in facilitating active interventions in a clinic environment, but they should not be considered in isolation as an effective approach.
